# Ultra-Brief Intervention for Problem Drinkers: Results from a Randomized Controlled Trial

**DOI:** 10.1371/journal.pone.0048003

**Published:** 2012-10-24

**Authors:** John A. Cunningham, Clayton Neighbors, Cameron Wild, Keith Humphreys

**Affiliations:** 1 Centre for Addiction and Mental Health, and University of Toronto, Toronto, Ontario, Canada; 2 Department of Psychology, University of Houston, Houston, Texas, United States of America; 3 Centre for Health Promotion Studies, School of Public Health, University of Alberta, Edmonton, Alberta, Canada; 4 Veterans Affairs and Stanford University Medical Centers, Stanford, California, United States of America; Centre for Addiction and Mental Health, Canada

## Abstract

**Background:**

There are a number of evidence-based, in-person clinical inteventions for problem drinkers, but most problem drinkers will never seek such treatments. Reaching the population of non-treatment seeking problem drinkers will require a different approach. Accordingly, this randomized clinical trial evaluated an intervention that has been validated in clinical settings and then modified into an ultra-brief format suitable for use as an indicated public health intervention (i.e., targeting the population of non-treatment seeking problem drinkers).

**Methodology/Principal Findings:**

Problem drinkers (N = 1767) completed a baseline population telephone survey and then were randomized to one of three conditions – a personalized feedback pamphlet condition, a control pamphlet condition, or a no intervention control condition. In the week after the baseline survey, households in the two pamphlet conditions were sent their respective interventions by postal mail addressed to ‘Check Your Drinking.’ Changes in drinking were assessed post intervention at three-month and six-month follow-ups. The follow-up rate was 86% at three-months and 76% at six-months. There was a small effect (*p* = .04) in one of three outcome variables (reduction in AUDIT-C, a composite measure of quantity and frequency of drinking) observed for the personalized feedback pamphlet compared to the no intervention control. No significant differences (*p*>.05) between groups were observed for the other two outcome variables – number of drinks consumed in the past seven days and highest number of drinks on one occasion.

**Conclusions/Significance:**

Based on the results of this study, we tentatively conclude that a brief intervention, modified to an ultra-brief, public health format can have a meaningful impact.

**Trial Registration:**

ClinicalTrials.gov NCT00688584.

## Introduction

Problem drinking is one of the three leading contributors to preventable burden of disease in high income countries [1], [2], [3], [4]. Consequently, there is considerable need to address the impact of drinking from a public health perspective. In their authoritative review of public health initiatives for problem drinking, Babor and colleagues [5] concluded that control initiatives such as taxation, limiting access, and drinking and driving laws have the best evidentiary base for demonstrating an impact on reducing alcohol consumption.

There is also substantial evidence that brief interventions can have a significant impact on problem drinking [6], [7], [8]. The difficulty, from a public health perspective, is how to deliver these proven interventions to a large enough group of problem drinkers in order to have a measurable impact on alcohol consumption at the population level because most problem drinkers will never access any type of treatment for their drinking [9]. One approach has been to promote the use of brief interventions by medical professionals in general practice settings [7], [8]. In fact, such interventions, although having less of a relevant evidence base than taxation, restricting availability, and drink driving legislation, nonetheless are regarded as having a sufficient evidence base to merit consideration as a population health intervention for problem drinking. [5]. Brief interventions, however, are procedurally different from the other three because these interventions usually require one on one interaction in primary care settings, an option which can be costly and is difficult to implement [10]. What other options for intervention exist [11]? There is substantial effort underway to establish and evaluate interventions situated on the Internet [12], [13], [14]. The Internet has the potential for wide spread impact because a growing number of people, including problem drinkers, will access health related information [15]. However, not all problem drinkers will actively seek out interventions on the Internet [16]. There is advantage to creating a range of different, research-validated interventions that have the potential for population level impact. Brief interventions by health professionals are one possible avenue. The Internet is another. By diversifying our options for helping problem drinkers in the general population, we have the potential of being able to impact the prevalence of alcohol problems. This is a worthy public health goal.

**Figure 1:**
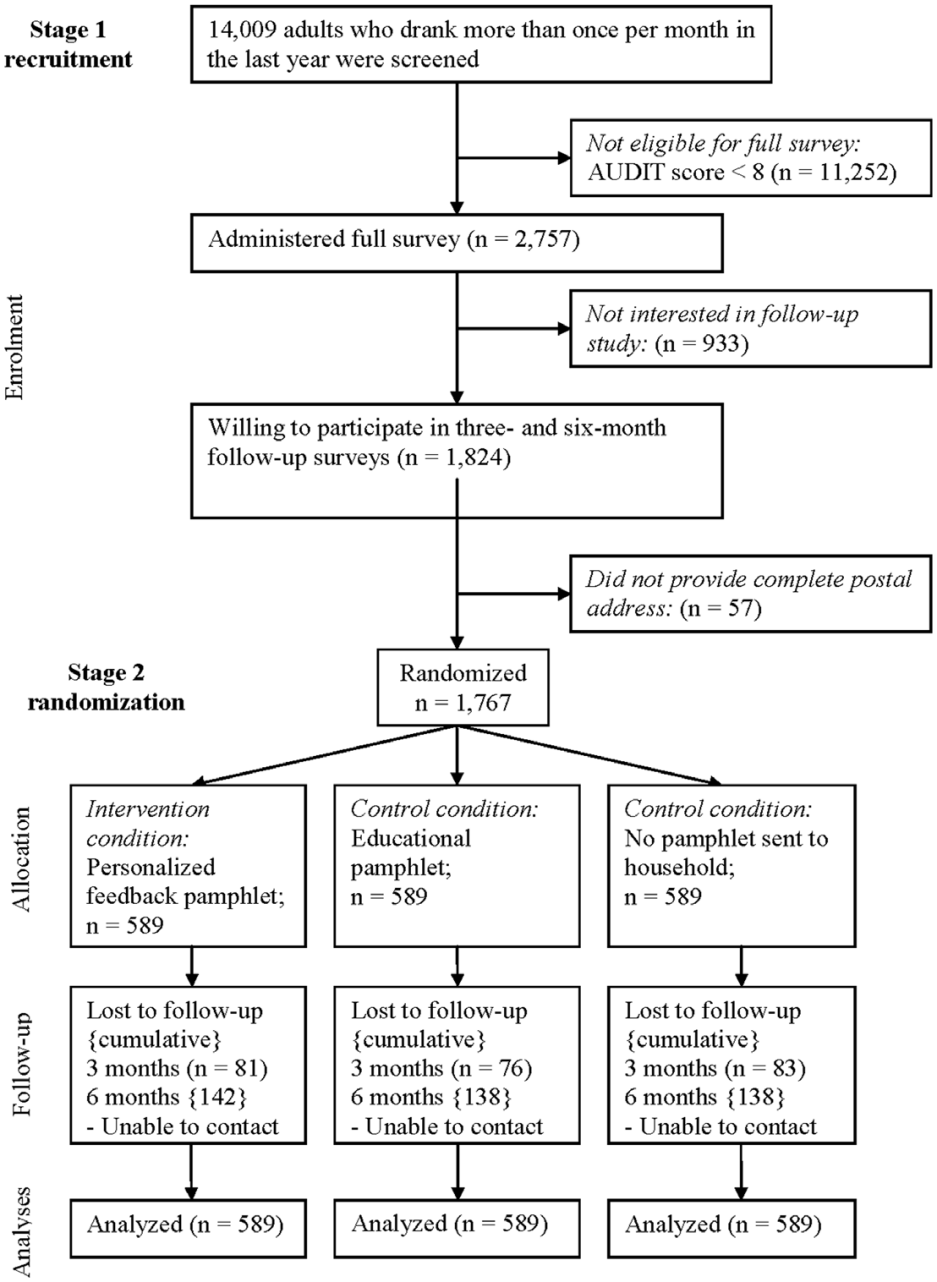
CONSORT diagram of participant recruitment.

Are there other alternative means to provide the core elements of a brief intervention in a manner which does not require one on one interaction? Preliminary studies have indicated that an ultra-brief intervention, in the form of a self-test personalized feedback pamphlet, may have an impact [17], [18]. However, research to-date has been limited to either short-term follow-up [17], by a research design that did not include randomization [19], or to evaluation among the subset of the drinking population who express interest in materials to help them evaluate their alcohol use [18]. It is important to evaluate the impact of ultra-brief interventions among all problem drinkers in order to establish if such interventions could be useful as a public health intervention (i.e., one that can be distributed widely and at low cost). This project evaluated the effectiveness of a pamphlet-based personalized feedback intervention for problem drinkers in the general population. The hypotheses regarding the effectiveness of the pamphlet-based intervention were: 1) respondents from households who receive the personalized feedback pamphlet-based intervention will display significantly improved drinking outcomes at three and six-month follow-ups as compared to respondents from households in the no intervention control condition relative to the null hypotheses that there will be no differences between conditions; and 2) respondents from households who receive the personalized feedback pamphlet-based intervention will display significantly improved drinking outcomes as compared to respondents from households who receive the control pamphlet relative to the null hypotheses that there will be no differences.

**Figure 2:**
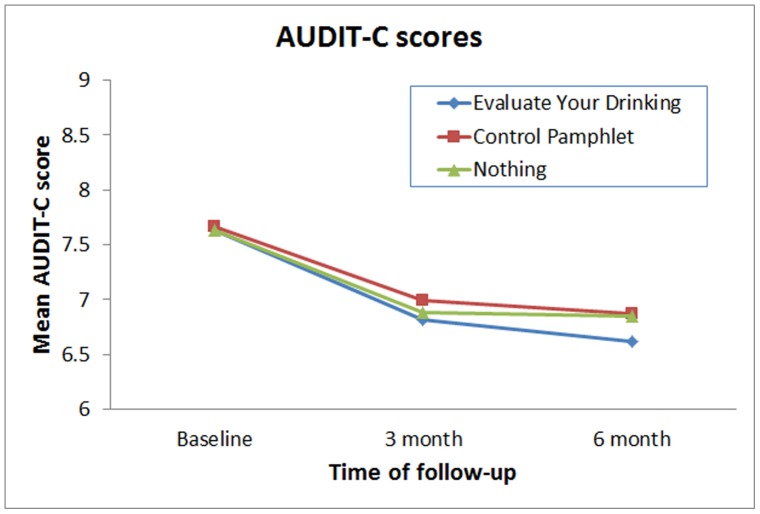
Mean AUDIT-C scores for participants in the three conditions across baseline, three-month, and six-month follow-ups.

**Table 1:** Mean (*SD*) drinking variables at baseline, three-, and six-month follow-up by study condition.

	Feedback condition			
Time	Intervention pamphlet(n = 589)	Control pamphlet(n = 589)	No pamphlet(n = 589)	*p*
	**Drinks in the past week**			
Baseline	12.3 (11.9)	13.0 (11.6)	11.6 (11.1)	
3-month	11.9 (11.4)	12.4 (11.6)	11.6 (11.1)	
6-month	11.8 (11.0)	12.2 (11.4)	11.9 (11.0)	
	**AUDIT-C score** a			
Baseline	7.7 (1.9)	7.7 (1.9)	7.7 (1.9)	
3-month	7.0 (2.4)	7.1 (2.2)	7.1 (2.3)	
6-month	6.8 (2.4)	7.0 (2.3)	7.0 (2.3)	.04*
	**Largest amount on one occasion**			
Baseline	9.6 (5.7)	9.6 (5.3)	9.2 (5.3)	
3-month	8.8 (5.4)	8.7 (5.2)	8.5 (5.0)	
6-month	8.5 (5.0)	8.5 (5.2)	8.5 (5.0)	

a The AUDIT-C is a composite measure of three drinking variables.

* slope of the AUDIT-C scores in the intervention pamphlet condition was significantly greater than the slope in the no pamphlet control condition from baseline to six-month follow-up, *p = *.04.

## Methods

The protocol for this trial is published [20] and the CONSORT checklist is available as supporting information; see Checklist S1 and Protocol S1.

### Ethics

The study was conducted in compliance with the Helsinki Declaration. Verbal consent was obtained from all participants as the initial contact was by telephone. Interviewers were trained in appropriate ethics procedures and telephone interviews were monitored by a supervisor to ensure adherence to training. This consent procedure and the conduct of the study were approved by the standing ethics review committee of the Centre for Addiction and Mental Health.

### Study Design and Population

A detailed research protocol is published elsewhere [20]. Briefly, households in a large metropolitan city were contacted as part of a random digit dialing survey. The interviewer asked to speak to the person (19 years or older – legal drinking age) in the household with the next birthday who also drank alcohol at least once per month. As part of the baseline survey, participants completed the Alcohol Use Disorders Identification Test (AUDIT) [21], [22]. Problem drinkers were identified as those with an AUDIT score of 8 or more. Problem drinkers were asked if they would be willing to take part in two more surveys – one in three months and the second in six months. Participants were told that the surveys would ask about their current drinking and no mention was made of the fact that they were selected for follow-up because they exhibited problematic alcohol use. They were told that they would be paid CDN $20 for completing each of these surveys. Participants agreeing to these additional surveys were also told, “the Centre for Addiction and Mental Health is in the process of mailing a safe-drinking pamphlet to some households in Toronto. I do not know if this pamphlet is being sent to your household, but if you do see it, the six-month follow-up survey will ask about your impressions of the materials.” Interviewers were blind to participants’ randomly assigned condition.

Participants who agreed to take part in the follow-up surveys were randomized into one of three conditions – sent a personalized feedback pamphlet, sent a control pamphlet which contained information on alcohol, or to a control condition sent nothing. Respondents were allocated to intervention and control conditions using a random number list generated for the study by the principal investigator. No stratification was employed, however, randomization was conducted by block in order to ensure equal number of participants per condition. Participants were assigned to condition by the project coordinator who sent out the intervention materials. The pamphlets were mailed in the week after the baseline survey. In order to mimic a mass mailing, the pamphlets were mailed to the household in an envelope addressed to, “Check your drinking,” rather than addressed to the individual.

### Description of Intervention Pamphlets

#### Evaluate your drinking pamphlet

This self-test pamphlet included basic elements of a personalized feedback intervention. The reader was first asked to record how many drinks they had consumed on each day of the past week and to sum the total (graphical standard drink definition was provided). The reader was then invited to compare their weekly drinking to that of males and females in the Canadian general population through pie charts depicting drinking in a typical week. The reader was then provided with information on the risk of experiencing negative consequences associated with different levels of alcohol consumption. The pamphlet concluded with a menu of different options they could choose with regard to their drinking. The pamphlet was professionally prepared in a multi-color format. A copy of the Evaluate your drinking pamphlet is available in Appendix S1.

#### Control pamphlet

The control pamphlet consisted of a standard educational pamphlet which contained information about alcohol consumption and safe consumption levels from an authoritative source (one of the ‘Do you know?’ series of pamphlets produced by the Centre for Addiction and Mental Health). A copy of the control pamphlet is available in Appendix S2.

### Main Outcome Variables

There were three primary outcome measures – number of standard drinks consumed in the past seven days, highest number of drinks on one occasion, and the AUDIT-C [23]. The latter is a composite measure consisting of three alcohol consumption measures (number of drinks per day, frequency of alcohol consumption, and frequency of consuming five or more drinks on one occasion – scored from 0 to 12 with a higher score indicating a more severe drinking problem). A standard drink was defined for participants (in Canada, a standard drink contains 13.6 grams of alcohol). All variables were examined for distributional properties and outliers were Winsorized (replacing any values beyond three standard deviations with the next highest value). The primary analyses employed an intent-to-treat approach. Missing values were handled using a last available value carried forward approach.

### Power Analysis

Based on our previous research with the intervention pamphlet, a 1% increase in explained variance was expected (a small effect size of f = 0.10; note – corresponded to a reduction of two drinks per week in the pilot study). We followed the convention that studies should be designed to have a statistical power of at least 80%, and that hypotheses be tested at the.05 level of significance. These specifications resulted in a final sample (required after attrition) of N = 390 in each condition (N = 1170 total).

### Analysis Plan

Analyses were conducted longitudinally with generalized linear mixed models (HLM) with random intercepts and fixed slopes. Intervention was specified using two dummy coded variables comparing participants from households sent the intervention pamphlet (intervention condition) to participants from households sent the control pamphlet (pamphlet control condition) and to those from households sent no pamphlet at all (no intervention control). Separate analyses were first conducted for each of the three outcome measures using the intent-to-treat approach described earlier and were then duplicated using only those participants with complete follow-up data.

## Results

A total of 14,009 participants were interviewed to identify 2757 with AUDIT scores of 8 or more. Of these, 1824 were willing to be followed-up and 1767 provided usable household postal addresses. Participants were recruited from the metropolitan Toronto area between December 2008 and November 2010. These 1767 participants were randomized to condition (Figure 1 provides a CONSORT diagram describing participant recruitment). Three-month follow-ups were completed for 1524 participants (86%; mean [SD] time to follow-up = 111.7 [26.8] days). Six-month follow-ups were completed for 1349 participants (76%; mean [SD] time to follow-up from baseline = 220.8 [40.0] days; only participants who completed the three-month follow-up were re-contacted at the six-month time point).

Bivariate comparisons found no differences in demographic and baseline drinking characteristics between experimental conditions. Of the 1767 participants, the mean (SD) age was 40.7 (14.7), 66.4% were male, 48.9% were married or living with a partner, 74.2% had some post-secondary education, and 74.3% were full or part time employed. The average (SD) AUDIT score was 12.1 (5.0).

Table 1 displays the means and standard deviations of the three outcome variables at baseline, three-month and six-month follow-ups. Only AUDIT-C scores displayed significant differences (*p*<.05) between conditions in the HLM analyses. Compared to participants in the no intervention control condition, participants in the intervention condition displayed a steeper reduction slope in their AUDIT-C scores from baseline to six-month follow-up. This difference was observed using an intent-to-treat approach, F(1, 3531) = 4.09, *p = *.043, or when only participants with complete follow-up data were employed, F(1, 2873) = 4.18, *p* = .041. Although in the predicted direction, differences in the slope for AUDIT-C scores between participants in the intervention condition were not significantly different from participants in the control pamphlet condition (*p*>.05). Figure 2 displays the pattern of results for the AUDIT-C scores between experimental conditions and across the three time points.

### Recall Receiving the Pamphlet

At the six-month follow-up, participants were asked if they recalled whether their household had received a pamphlet from CAMH. In the intervention pamphlet condition, 38% recalled receiving a pamphlet, while 34% of participants in the control pamphlet condition recalled receiving a pamphlet and 6.9% of participants in the households which were not sent a pamphlet reported receiving one. Marginally more participants in the intervention pamphlet condition recalled their household receiving the correct pamphlet (20.8% recalled receiving the Evaluate Your Drinking pamphlet) as compared to participants from households in the control pamphlet condition (15.3%, Fisher’s Exact test, *p* = .037).

## Discussion

There was tentative support for the impact of the pamphlet based personalized feedback. On one of the three outcome variables, AUDIT-C scores, participants from households who received the intervention pamphlet (Evaluate Your Drinking) reported a greater reduction in AUDIT-C scores over time (three and six month follow-ups) as compared to participants from households who did not receive any pamphlet. This finding was observed both when missing data was replaced by carrying forward the last completed value and when only participants with complete follow-up data were employed in the analyses. However, while in the predicted direction, participants whose households were in the intervention pamphlet condition did not display greater reductions in AUDIT-C scores as compared to participants whose households were sent the control pamphlet that contained educational information about alcohol but no personalized feedback exercise. In addition, differences between condition for the other two outcome variables – number of drinks in the past week and highest number of drinks on one occasion – were not statistically significant (*p*>.05).

A conservative interpretation of these results is that the personalized feedback intervention pamphlet failed to demonstrate reasonable evidence of an impact on drinking. Given that the trial was powered to detect a small effect size, and sample sizes were estimated using results from previous trials employing the same intervention, it is reasonable to conclude that this was a negative trial and that the ultra-brief intervention, when tested in a situation that mimics a real world, public health distribution of this pamphlet, does not have an effect on drinking.

Is there another, justifiable way to interpret these results? The Evaluate Your Drinking pamphlet is a very brief, self-administered, paper version of the personalized feedback intervention. The pamphlet can be distributed widely and at low cost – important hallmarks when considering the utility of public health interventions. The study design was set up to mimic a public health mass distribution of educational materials about drinking. That is, the pamphlet was delivered to the household rather than addressed to the participant. The participants in the trial were problem drinkers who agreed to be followed-up. There was no pre-screening to identify participants who would be interested in these materials or who were already concerned about their drinking. In addition, the impact of this very minimal intervention had to be observed over and above the documented impact associated with just participating in a structured interview about one’s own drinking [24], [25]. Given these design elements in this trial, it is encouraging to see any impact of the pamphlet at all, even if on only one of the three outcome variables and just in comparison to participants from the no intervention condition.

There were several limitations to this trial. Follow-up rates were only minimally acceptable (76% with complete data). In addition, the timing of the follow-ups was not strictly adhered to and a minority of participants were interviewed long after their scheduled follow-up dates. Further, the results relied on self-report data alone. Despite these limitations, we are fairly confident of the reliability of the results given that the study was set up to minimize the social desirability associated with being in an intervention condition as participants were essentially blind to the fact that they had been randomized at all. We conclude that the results of this study provide tentative support for the impact of this ultra-brief intervention and merit systematic replication that incorporates accurate sample size estimates based on the results of this trial as well as a more tightly controlled follow-up interview schedule.

## Supporting Information

Checklist S1 **Consort checklist.**
(DOCX)

Protocol S1 **Publication of Study Protocol.**
(PDF)

Appendix S1 **Copy of Evaluate Your Drinking Pamphlet.**
(TIF)

Appendix S2 **Copy of Control Pamphlet.**
(TIF)
