# Supplementary material for: Ultra-Brief Intervention for Problem Drinkers: Results from a Randomized Controlled Trial
Source: PLoS One. 2012 Oct 24;7(10):e48003. doi: 10.1371/journal.pone.0048003 (PMC3480504; doi:10.1371/journal.pone.0048003)
Supplement: Protocol S1 — Publication of Study Protocol. (PDF) [file pone.0048003.s002.pdf]

BMC Public Health

BioMed Central

Study protocol

Open Access

Ultra-brief intervention for problem drinkers: research protocol

John A Cunningham*[1], Clayton Neighbors[2], Cameron Wild[3] and Keith Humphreys[4]

Address: [1]Centre for Addiction and Mental Health, 33 Russell Street, Toronto, Ontario, M5S 2S1, Canada, [2]Department of Psychiatry and Behavior Sciences, University of Washington, 1100 NE 45th St., Suite 300, Box 354944, Seattle, WA, 98195, USA, [3]Centre for Health Promotion Studies, School of Public Health, University of Alberta, 7-30 University Terrace, Edmonton, Alberta, T6G 2H1, Canada and [4]Veterans Affairs and Stanford University Medical Centers, 401 N. Quarry Road, Room C-305, Stanford, CA, 94305-5717, USA

Email: John A Cunningham* - john_cunningham@camh.net; Clayton Neighbors - claytonn@u.washington.edu; Cameron Wild - cam.wild@ualberta.ca; Keith Humphreys - knh@stanford.edu

* Corresponding author

Published: 26 August 2008

BMC Public Health 2008, **8**:298 doi:10.1186/1471-2458-8-298

Received: 30 July 2008

Accepted: 26 August 2008

This article is available from: http://www.biomedcentral.com/1471-2458/8/298

© 2008 Cunningham et al; licensee BioMed Central Ltd.

This is an Open Access article distributed under the terms of the Creative Commons Attribution License (http://creativecommons.org/licenses/by/2.0), which permits unrestricted use, distribution, and reproduction in any medium, provided the original work is properly cited.

Abstract

Background: Helping the large number of problem drinkers who will never seek treatment is a challenging issue. Public health initiatives employing educational materials or mass media campaigns have met with mixed success. However, clinical research has developed effective brief interventions to help problem drinkers. This project will employ an intervention that has been validated in clinical settings and then modified into an ultra-brief format suitable for use as a public health intervention. The major objective of this study is to conduct a randomized controlled trial to establish the effectiveness of an ultra-brief, personalized feedback intervention for problem drinkers.

Methods/design: Problem drinkers recruited on a baseline population telephone survey conducted in a major metropolitan city in Canada will be randomized to one of three conditions – a personalized feedback pamphlet condition, a control pamphlet condition, or a no intervention control condition. In the week after the baseline survey, households in the two pamphlet conditions will be sent their respective pamphlets. Changes in drinking will be assessed post intervention at three-month and six-month follow-ups. Drinking outcomes will be compared between experimental conditions using Structural Equation Modeling. The primary hypothesis is that problem drinkers from households who receive the personalized feedback pamphlet intervention will display significantly improved drinking outcomes at three and six-month follow-ups as compared to problem drinkers from households in the no intervention control condition. Secondary hypotheses will test the impact of the intervention on help seeking, and explore the mediating or moderating role of perceived drinking norms, perceived alcohol risks and the problem drinker's social reasons for drinking.

Discussion: This trial will provide information on the effectiveness of a pamphlet-based personalized feedback intervention for problem drinkers in a community setting.

Trial registration: ClinicalTrials.gov registration #NCT00688584.

Background

The ratio of problem drinkers to those seriously dependent on alcohol is about 4:1 [1]. As Cahalan [2] has noted, "clinically defined alcoholics constitute only a relatively small proportion of those whose drinking creates significant problems for themselves and society" (p. 363). The majority of these problem drinkers, while being at risk for health problems and other psychosocial consequences [3], will never access any treatment services. (estimated ratio of treated to untreated problem drinkers ranges from 1:3 to 1:14 in Canada and the United States) [4-9]. Many individuals with drinking problems do not approach alcohol treatment facilities, often because of stigma, embarrassment or because they don't think of their drinking as a problem [5,10-12]. Further, when asked why they have not sought formal help or treatment, problem drinkers overwhelmingly indicate that they "want to change on their own" [10,13]. The present study proposes to test the effects of a self-change intervention that circumvents some of the barriers of traditional treatment by allowing problem drinkers to work on their alcohol problems on their own in private without approaching alcohol treatment facilities.

In 1990, the Institute of Medicine [1] recommended a broadening of the range of services for people with alcohol problems. Providing self-help interventions to individuals who have not accessed treatment services is one way to accomplish this important public health goal and ensure that a broader range of services is available. Although such interventions might only have a limited impact on an individual level, they can still be beneficial as part of a continuum of care for individuals unlikely to enter formal alcohol treatment programs [1,14,15]. Further, when viewed from a public health perspective [16], minimal interventions have the potential for a significant population-level impact as such interventions are low cost and can be provided to a large number of drinkers. Public health impact is typically conceptualized as the reach of the intervention X efficacy per unit cost [17]. The low cost and potential for broad reach of self-help interventions, coupled with the high base rate of the behavior suggests that such interventions can have a significant public health impact.

Public Health 'Educational' Initiatives to Reduce Problem Drinking

As problem drinking is one of the five leading contributors to the global burden of disease [18], there is considerable need to address the impact of drinking from a public health perspective. In their authoritative review of public health initiatives for problem drinking, Babor and colleagues [19] concluded that policy initiatives such as taxation, limiting access, and drinking and driving laws have the best research base for demonstrating an impact on reducing alcohol consumption. Educational initiatives were judged to be ineffective. Public education initiatives are distinct from school education programs and cover the domain of public marketing campaigns such as responsible drinking advertisements, banner advertisements and other media initiatives. Babor and colleagues concluded that there was no evidence for such public educational initiatives having a measurable impact on drinking. Other reviews on this topic have been less negative [20], although none make the claim that such campaigns can lead to reductions in alcohol consumption. Rather, the role of these educational campaigns is to cause changes in attitudes towards drinking and to provide public support for control initiatives, such as taxation and drinking-driving laws, so that these control initiatives can cause reductions in alcohol consumption.

Is the conclusion that public health educational initiatives cannot directly cause reductions in alcohol consumption a definitive one? We do not believe so. Our argument is that educational initiatives would benefit from looking at the brief intervention literature. As will be outlined below, there is substantial evidence that brief alcohol interventions can have a significant impact on problem drinking [21,22]. The difficulty, from a public health perspective, is how to deliver these efficacious interventions to a large enough group of problem drinkers in order to have a measurable impact on the population level of alcohol consumption. This high level of impact has been demonstrated within 'special' populations, such as college drinkers [23]. However, the challenge becomes greater when we consider ways to impact on the general population of problem drinkers in entire countries such as Canada and the United States because most problem drinkers will never access any type of treatment for their drinking [8,9]. One approach has been to promote the use of brief interventions by medical professionals in general practice settings [24,25]. Unfortunately, many drinkers in the general population may never receive a preventive alcohol intervention in the context of primary health care [26,27]. What other options for intervention exist? There is substantial effort underway to establish and evaluate interventions situated on the Internet [28]. The Internet has the potential for wide spread impact because a growing number of people, including problem drinkers, will access health related information [29]. However, although the potential is there, not all problem drinkers will actively seek out interventions on the Internet [30,31]. We argue that there is advantage to creating a range of different, research validated interventions that have the potential for population level impact. Brief interventions by health professionals are one possible avenue. The Internet is another. The current study seeks to evaluate a third, an ultra-brief intervention in the form a self-test personalized feedback pamphlet. By diversifying our options for help-

ing problem drinkers in the general population, we have the potential of being able to impact the prevalence of alcohol problems. This is a worthy public health goal.

Efficacy Trials of Personalized Alcohol Feedback Paper and Pencil Self-Change Interventions

Personalized alcohol feedback interventions are designed to increase motivation for behavior change [32,33]. Such materials provide normative feedback to individuals – providing a personalized summary of an individual's drinking and comparing it to the consumption of the average male or female in the general population. Normative feedback is theorized to promote change in alcohol use because many heavy drinkers overestimate the consumption of others. Consequently, normative feedback acts as a powerful source of social comparison, motivating heavy drinkers to re-evaluate their consumption patterns [34].

Personalized alcohol feedback has been found to promote behavior change in drinkers [32,35-45]. In the college student literature brief interventions utilizing relevant personalized feedback, as opposed to more general social norms campaigns, have consistently been found efficacious whether delivered in-person, by computer, or mail [23,46]. In particular, mailed feedback, similar to the intervention in this study has been found effective in reducing drinking and preventing onset and escalation of drinking in college students [32,35-37,45,47]. Analogous mailed feedback has also been effective in reducing symptoms of depression [48]. A recent meta-analysis by Carey and colleagues [49] also identified normative feedback as an effective intervention to reduce problem drinking in college students.

Personalized alcohol feedback interventions are particularly well suited for pamphlet-based delivery. This is because such interventions can be translated into a simple, self-test format. The other advantage of personalized feedback interventions is their brevity – both as far as the assessment required and in the time required to complete them. Such features are important as pamphlets sent directly to households have to attract the readers' attention quickly and be easy to complete. Neighbors and colleagues [44] have also demonstrated that even the most minimal of normative interventions can have an impact on drinking at a six-month follow-up among college students, lending confidence to the possibility of creating an effective, ultra-brief personalized feedback pamphlet. Further, a recent study conducted by this research group has demonstrated an impact on drinking by our ultra-brief, personalized feedback pamphlet at a six-month follow-up, providing evidence for a sustained effect of this approach (study described in more detail in the Preliminary Studies section of this application) [38].

Do personalized alcohol feedback interventions work because they modify perceived drinking norms?

Several researchers have applied self-regulation theory [50] as a model to help explain the impact of personalized feedback interventions [32,51,52]. By overestimating the prevalence of heavy consumption among peers, heavy drinkers are thought to view their own behavior as normative rather than abnormal or inappropriate. Personalized normative feedback is theorized to develop discrepancy in the recipient by providing information showing that their own drinking is not normative [41,45,53]. Alerting heavy drinkers to the fact that their own drinking is abnormal (i.e., developing discrepancy) is theorized to result in problem recognition and may instigate behavior change [44,51,54]. If this process does mediate the impact of normative feedback interventions then, for the intervention to work, it must modify recipients' perceptions of how much others actually do drink. The modification of these perceptions would then lead to an increase in perceptions of discrepancy between their own and others' drinking. Because of the importance of understanding why normative feedback interventions work, the current study will include perceived drinking norms as a hypothesized mediator of the impact of the personalized feedback pamphlet intervention.

Does Perceived Risk Mediate the Effects of Safe Drinking Interventions?

The motivational impact of perceived risk is clearly shown by considering four cognitive theories of health protective behavior. In particular, the health belief model [55], protection motivation theory [56], the theory of reasoned action [57], and subjective expected utility theory [58] are all identical insofar as they each assume "that anticipation of a negative health outcome and the desire to avoid this outcome or reduce its impact creates motivation for self-protection" [p. 234, [59]]. We have also conducted correlational research exploring why some drinkers perceive risk associated with their drinking while others do not. These studies concluded that there is a need to consider perceived risk as well as objective problem status when designing and evaluating interventions to help heavy drinkers [60]. One of the studies described in the Preliminary Studies section below also indicates the mediating role that perceived risk may play in drinkers' reactions to safe drinking interventions [39].

Why might perceived risk mediate the impact of personalized feedback interventions? As was discussed earlier, such interventions provide normative feedback to respondents, comparing their drinking to others in the general population. As many heavy drinkers overestimate how much others drink, this normative information often comes as a surprise to them, allowing them to make the social comparison that they drink more than others [34]. According

to cognitive theories of health protective behavior, this information leads to changes in health behavior because it results in an increase in respondents' perceived vulnerability. The recipient recognizes that he or she is engaged in a risky health behavior (i.e., drinking more than others). This increase in perceived risk then motivates respondents to reduce their drinking. The current study will test whether changes in perceived risk mediate reductions in drinking. Although not necessarily contradictory, the mediator hypotheses of perceived drinking norms and of perceived risk come from different theories of change. This study will measure both these constructs and evaluate their validity as potential mediators of the impact of personalized feedback interventions. Secondary analyses will also explore the possible interrelationship between perceived risk and drinking norms as hypothesized mediators of the impact of the ultra-brief intervention.

One alternative explanation of the role of perceived risk is that it may also act as a modifier of respondents' reactions to safe drinking interventions. That is, problem drinkers may vary in their perceived risk about drinking at baseline, before they receive the intervention. Those problem drinkers who perceive some risk with regard to their drinking may utilize the intervention and reduce their drinking. Those problem drinkers who perceive no risk associated with their drinking may disregard the intervention materials and thus display no reduction in drinking.

Is normative feedback more important for problem drinkers who drink for social reasons?

Using the theoretical underpinnings of expectancy research [61,62] and the concept of social drinking motives [63], Neighbors and colleagues [44] suggested that the fact that people vary in the extent to which they drink for social reasons might have matching implications for normative feedback interventions. By social reasons, Neighbors and colleagues (2004) were referring to drinking because of anticipated social positive reinforcement. Normative feedback may be more important for people who drink primarily for social reasons as compared to those who don't because people who drink for social reasons may anticipate more social benefits from their drinking. Neighbors et al. [44] found evidence for this moderating effect on the impact of normative feedback information in a sample of college students. The present study will explore the moderating role of social reasons for drinking in order to identify those who might benefit most from pamphlet-based personalized feedback interventions.

Preliminary Studies

There are two previous studies that have employed this same personalized feedback pamphlet.

Preliminary Study # 1 – Pamphlet Personalized Alcohol Feedback Trial

This pilot study demonstrated the potential of the ultra-brief intervention for problem drinkers to be used in the present study [39]. The "Evaluate Your Drinking" pamphlet contains a self-test that allows the reader to compare his or her personal drinking to that of other Canadians (see Methods section for a more detailed description of this pamphlet).

The pamphlet was sent by unaddressed ad mail to households, randomized by block to receive or not receive the brief intervention. A random digit dialing telephone survey was conducted in the following month to assess differences in drinking between experimental conditions. The primary result was a 3-way interaction between pamphlet condition, perceived risk, and problem drinking status. The pattern of means indicated that the manner in which perceived risk mediated the impact of pamphlet conditions differed, depending on whether respondents met objective criteria for problem drinking. For non-problem drinkers there was no significant difference between intervention conditions, irrespective of whether the person perceived his or her drinking to be of some or no risk. However, among problem drinkers who perceived no risk, there was a trend for those who received the intervention to drink more as compared to those who did not receive the intervention ($p < .06$). In contrast, among problem drinkers who perceived some risk, respondents who received the pamphlet were drinking less than those who did not ($p < .05$).

The results of this trial indicated that perceived risk might act as either a mediator or a moderator of respondents' reactions to safe drinking interventions. This finding is important to confirm because it points to one of the factors that may differentiate those who continue problem drinking from those who reduce their drinking. However, the post-test only design employed in this research did not allow causal statements to be made regarding the effects of respondents' perceived risk because this variable, along with all others, was assessed after the intervention was administered. Thus, there was no way of knowing whether those with greater perceived risk about alcohol consumption at the one-month follow-up experienced an increase in their perceived risk after receiving the intervention or had higher perceived risk scores already at baseline. To address this issue, the current study will conduct an appropriate evaluation of the role of perceived risk, assessing it at baseline as well as at the three-month follow-up, and then evaluating the potential mediating or moderating effect of perceived risk on problem drinkers' drinking outcomes at six-month follow-up.

It is also important to conduct an appropriate evaluation of the role of perceived risk because of the possibility indicated in this pilot study that respondents with no perceived risk might react negatively to receiving the pamphlet. This post-test only pilot study can only be taken as the most preliminary of evidence that those respondents without any perceived vulnerability with respect to their drinking might react negatively to the pamphlet by drinking more. However, we feel that the possible 'boomerang' effect of this ultra-brief intervention among problem drinkers with no perceived risk is an important one to investigate. This is because confirmation of this pattern of results would indicate the need to target interventions, such as this normative feedback pamphlet, to problem drinkers who are concerned about their drinking. Thus, we feel that the potential benefits of conducting this test outweigh the ethical risks associated with conducting a trial where there is the potential of a small negative impact among some participants.

Preliminary Study # 2 – Replicating the impact of the ultra-brief intervention

Wild and colleagues conducted a trial that further supported the ultra-brief pamphlet intervention [38]. Drinkers included in a general population telephone survey, who indicated that they were hypothetically interested in receiving self-help materials, were recruited through a general population telephone survey (n = 1720). Respondents who agreed to a six-month follow-up were randomly assigned to receive or not receive the same pamphlet intervention that was used in Study # 1. Residualized change score analysis found that, among respondents who met criteria for problem drinking at baseline, the intervention group showed a 10% reduction in per-occasion binge drinking (i.e., consuming 5 or more standard drinks per occasion), compared to controls ($p < .01$). Notably, we observed no iatrogenic effects of providing the pamphlet to drinkers who did not meet criteria for hazardous drinking at baseline, i.e., no escalation of drinking among no-problem drinkers after receiving the ultra-brief intervention.

Although the results of this study are encouraging, only respondents who were interested in receiving self-help materials were recruited for the intervention. For the present study, we intend to evaluate the impact of the ultra-brief pamphlet intervention, whether the respondent is specifically interested in receiving self-help materials or not (note: while still maintaining fully informed consent). This is a challenging undertaking but we feel that such a goal is essential if we are to evaluate the effectiveness of the intervention within the same setting that interventions, such as the one to be used in the present study, would be employed in real life. It should also be noted that only recruiting respondents who say they are interested in self-help materials has the additional limitation of excluding many respondents who perceive no risk associated with their drinking. In Preliminary Study # 1, 76% of the respondents who perceived no risk associated with their drinking also said they were not interested in self-help materials. Thus, we must employ a design that recruits all problem drinking respondents, whether interested in self-help materials or not, in order to fully explore the public health impact of this intervention approach, and to conduct an adequate test of the potential role of perceived vulnerability as a mediator and a moderator of the intervention effects. One final limitation of the Wild et al. study was that there is no way to tell whether the observed impact on drinking was due to the content of the personalized feedback pamphlet or simply because the respondent received any alcohol-related pamphlet at all. Thus, the current study incorporates a second control condition in which the households of a randomized third of the participants will receive a popular educational pamphlet on alcohol that contains no personalized feedback content.

What is the principal research objective?

The principal research objective is to evaluate the efficacy of a pamphlet-based self-help intervention among problem drinkers in the general population. The development of an effective, research-based pamphlet of this type is important because of its potential for use in public health initiatives where low-cost and wide distribution are key considerations. In order to mimic the use of a personalized feedback pamphlet in a public health initiative, the pamphlet will be sent unaddressed to households rather than to specific individuals. In addition, because it is important to determine whether it is the content of the personalized feedback pamphlet or just the receipt of any pamphlet that leads to reductions in drinking, a control pamphlet condition will be included in the study.

Methods/design

Aim

The proposed research will evaluate the efficacy of a pamphlet-based personalized feedback intervention for problem drinkers in the general population.

The hypotheses regarding the efficacy of the pamphlet-based intervention are:

Hypothesis 1: Respondents from households who receive the personalized feedback pamphlet-based intervention will display significantly improved drinking outcomes at three and six-month follow-ups as compared to respondents from households in the no intervention control condition.

Hypothesis 2: Respondents from households who receive the personalized feedback pamphlet-based intervention will display significantly improved drinking outcomes as compared to respondents from households who receive the control pamphlet.

In addition, two mediator hypotheses will be tested:

Hypothesis 3: Respondents who receive the intervention and reduce their estimates about how much others drink between baseline and three-month follow-up will display significantly improved drinking outcomes at six-month follow-up as compared to respondents who receive the intervention but experience no decrease in their perceived drinking norms.

Hypothesis 4: Respondents who receive the intervention and experience an increase in their perceived vulnerability to experience harm because of their alcohol consumption between baseline and three-month follow-up will display significantly improved drinking outcomes at six-month follow-up as compared to respondents who receive the intervention but experience no increase in their perceived risk.

Finally, two moderator hypotheses will be tested:

Hypothesis 5: The impact of the intervention will be greater among drinkers who believe that they are personally vulnerable to negative outcomes at baseline assessment, compared to drinkers who do not believe that they are personally 'at risk' before receiving the intervention.

Hypothesis 6: Respondents who drink for social reasons will be more likely to reduce their drinking as a result of the intervention as compared to respondents who do not drink for social reasons.

Design

In an initial telephone interview, sociodemographic information will be collected. Current drinkers will complete a standardized epidemiological assessment of problem drinking and alcohol consumption, and will answer items to assess their perceived drinking norms, their perceived risk regarding drinking and their social reasons for drinking. Respondents identified as problem drinkers will be asked if they are willing to participate in a three-month and a six-month follow-up. Respondents will be offered a $20 honorarium for completion of each of the three-month and six-month follow-ups. Verbal informed consent will be gathered and respondents will be told that some households will be receiving a pamphlet. Problem drinking will be defined as a score of eight or more on the Alcohol Use Disorders Identification Test (AUDIT) [64,65]. The initial telephone survey will be conducted with a random sample of respondents from households within a major metropolitan city in Canada. One current drinker 19 years or older (legal drinking age in Ontario, Canada) will be selected to participate from each household by soliciting participation from the adult in the household who had the most recent birthday who also drinks alcohol at least once per month. All households with respondents agreeing to participate in the follow-up interviews will be randomized into three groups – personalized feedback pamphlet condition, control pamphlet condition and no intervention control condition. In the week after the baseline survey, all households that contain respondents in the two pamphlet conditions will be sent their respective pamphlets. Three months after the intervention mailing, respondents who agree to the follow-ups will be administered a second telephone interview assessing the same drinking and mediator terms as the baseline survey (modified to refer to the past three months). Similar drinking outcome measures will be made six months after the intervention mailing with the items framed to refer to the past three-months. In order to ensure that all study participants receive some form of intervention, households in the no intervention control condition will be sent the personalized feedback intervention pamphlet after the six-month follow-up.

Rationale for the choice of study design

Why do we believe that the proposed study design is the best for testing the impact of this personalized feedback pamphlet? The intent of this project is to evaluate an ultra-brief intervention for problem drinkers that can be used in public health initiatives. As such, it is important that the intervention pamphlet be evaluated in a setting that mimics how it will be used. Thus, the pamphlets will be sent addressed to the household rather than addressed to the respondents because public health initiatives are often non-specific in their target recipients. The proposed research design has the advantage of allowing the pamphlet to be sent to all households in the intervention condition, irrespective of whether any of the respondents are specifically interested in receiving self-help materials. In order to conduct a study where intervention materials are sent directly to the person by name, for ethical reasons the respondent must at least state that he or she is hypothetically interested in receiving such materials. A study in which materials are sent unsolicited to households has been judged ethical because receiving such materials unsolicited (and unaddressed to a specific individual) is not an unusual occurrence given that public health initiatives are ongoing in the Toronto metropolitan area. However, informed consent is given because all respondents are told about the possibility of their household receiving the pamphlet. Further, it is important to be able to send materials to all households because interest in self-help materials covaries with respondents' perceived risk. In the

pilot study described above, 76% of problem drinkers who perceived no risk associated with their drinking were not interested in self-help materials. This group comprised 32% of all problem drinkers in the sample. Thus, although the proposed method reduces the power of the intervention because not all respondents will see the pamphlet, it is still the best design for our purposes because it allows recruiting some problem drinkers with no perceived risk regarding their alcohol consumption. Studying the reactions of problem drinkers who do not perceive any risk associated with their drinking to intervention materials is of great importance because results from the pilot study for this proposal (Study 1 in the preliminary studies section) indicated that this group could react to the intervention materials by drinking more. Although the results of Study 1 were not conclusive because of its post-test only design and the fact that this 'boomerang' effect in reaction to the materials was a trend rather than a statistically significant result, we feel that the study of these types of unintentional effects is essential. The personalized feedback pamphlet is intended for wide distribution in the general public. If we confirm that this ultra-brief intervention can have a negative impact on some sub-groups of problem drinkers then this finding would speak to the need to target the intervention to only those drinkers who already voice some concern regarding their drinking. As there is insufficient evidence to-date that could confirm or refute this hypothesis, we feel that the need to test this hypothesis appropriately outweighs the potential ethical dilemma of causing a small unintended increase in alcohol consumption among a population of drinkers who are not seeking treatment and who meet criteria for a rather liberal definition of problem drinking (AUDIT score of 8 or more).

Informed Consent Procedure

The study was approved by the standing ethics review committee of the Centre for Addiction and Mental Health. Telephone calls will be made by trained interviewers from the Institute of Social Research at York University (Toronto). The interviewers will introduce themselves, indicate they are calling from York University and that they are calling on behalf of the Centre for Addiction and Mental Health, which is conducting research on drinking. They will determine the number of monthly drinking adults in the household, randomly select one of them (according to most recent birthday) and before they start the interview they will read the following script:

> "I would like to assure you that all information you provide, including your answers, identity, and any other information will remain completely confidential. You do not have to answer any questions you do not want to and if you decide to stop the interview, and wish us to do so, we will destroy all the information you have given us. On average, the interview will take about 15 minutes. Just to let you know, from time to time my supervisor may listen in to make sure we are doing the research correctly. The survey is voluntary, but your participation is very important if the results are to be accurate. Is now a good time to start the interview?"

At the end of the baseline survey, all problem drinkers (AUDIT ≥ 8) will be asked to participate in two, 15 minute surveys, the first in about three months time and the second in six months time. Participants will be offered $20 for the completion of each of the three-month and six-month follow-ups. Potential respondents will be told that these surveys will ask about their current drinking. Further, they will be informed that "the Centre for Addiction and Mental Health is in the process of mailing a safe-drinking pamphlet to some households in Toronto. I do not know if this pamphlet is being sent to your household, but if you do see it, the six-month follow-up survey will ask about your impressions of the materials." Respondents who agree to the follow-up will provide their name, address and telephone number. Because the baseline interview contact is made by telephone, verbal agreement to participate in the study will act as providing informed consent. Respondents will also be informed of the request to provide a collateral on the six-month follow-up at this time.

The Personalized Feedback Pamphlet ("Evaluate Your Drinking")

The "Evaluate Your Drinking" self-test pamphlet was modeled after the Drinker's Check-up [32,40,66] and the Fostering Self-Change intervention [33]. The pamphlet starts with an encouragement for the reader to evaluate his or her own drinking. The reader is then asked to record his or her drinking for each day of a typical week and to sum this information to calculate the number of drinks usually consumed per week (a 'standard drinks' chart is provided to help the reader). Next, information on the drinking patterns of males and females in the general Canadian population is provided. The reader is encouraged to compare his or her personal drinking to that of other Canadians and a graph is presented of the likelihood of adverse effects associated with different levels of consumption. The pamphlet concludes with a menu of options and encourages those readers who are concerned about their drinking to take the next step towards changing their alcohol consumption. Incorporated in this menu are low-risk drinking guidelines and a toll-free telephone number for individuals who would like to call to receive a free referral to a local treatment agency. To enhance impact and readability, the pamphlet was professionally produced in a multi-color, glossy format. The pamphlet was modeled after research-validated personalized feedback interven-

tions and provides an easily completed normative feedback self-test for the reader in an attractive and eye-catching format [see Additional file 1].

Control Condition Pamphlet ("Do You Know ... Alcohol")

The control pamphlet called 'Do you know .... Alcohol' is disseminated by the Centre for Addiction and Mental Health (CAMH). This pamphlet provides good quality information about alcohol, consequences of its misuse and safe drinking guidelines (same guidelines as are used in the personalized feedback pamphlet). The "Do you know" pamphlet is one of the most popular distributed by CAMH and is a high quality pamphlet that is a good example of educational materials disseminated about alcohol [see Additional file 2].

Content of the Baseline Survey

(1) The Alcohol Use Disorders Identification Test will be used to measure level of alcohol consumption and severity of alcohol problems (a score of 8 or more on the AUDIT indicates a past 12 month alcohol problem) [64,65]. The AUDIT has been validated for use in telephone surveys [67]. Usual quantity of drinking will be assessed as a continuous variable. An additional item will ask the highest number of drinks the respondent recalled having on any one occasion in the past three months. Finally, respondents' drinking will also be assessed by asking the number of drinks consumed on each day of the last week.

(2) Six items assessing whether in the past 12 months alcohol had a harmful effect on respondents' (1) friendships/social life, (2) physical health, (3) outlook on life (happiness), (4) home life or marriage, (5) work, studies, or employment opportunities, or (6) financial position [68].

(3) Perceived drinking norms will be measured using a modified version of the Drinking Norms Rating Form [69]. Respondents will be asked how often they think a typical person their age and gender drinks and how often they consume five or more drinks on one occasion (same category response options as AUDIT). Further, respondents will be asked to estimate how much the typical person their age and gender usually drinks on one occasion.

(4) Perceived risk for drinking-related problems will be assessed using a six-item scale with questions about perceived vulnerability to harm from the drinkers' own perspectives [70].

(5) Following the procedure used by Neighbors [44], social reasons for drinking will be measured using the Social Rewards subscale of the Drinking Motives Questionnaire [63] a five item scale that asks respondents how often they are motivated to drink for positive social outcomes. Also, social outcomes expectancies and subjective evaluations of social effects of alcohol will be assessed using the sociability subscales of the Comprehensive Effects of Alcohol Scale [71].

(6) Formal addictions treatment utilization will be measured using a single item from the National Longitudinal Alcohol Epidemiological Survey [72]; "Have you ever gone anywhere or seen anyone for a reason that was related in any way to your drinking – a physician, counselor, Alcoholics Anonymous, or any other community agency or professional? Include help for combined alcohol and other drug use if alcohol was the major problem for which you sought help."

(7) A series of demographic characteristics will be assessed: age, gender, ethnicity, marital status, education, and employment status.

Problem drinkers (AUDIT score of 8 or more) will be recruited to participate in a three-month and a six-month follow-up telephone survey.

Three-month Follow-up Survey

The three-month follow-up will occur roughly three-months after the intervention pamphlet is sent (or equivalent time for those in the control condition).

(1) Drinking in the last three months will be assessed using the same five items employed on the baseline survey, the first four framed for the past three months (frequency of consumption, drinks per occasion, frequency of 5+ consumption, highest number of drinks on one occasion) and the last asking how much the person drank on each day of the last week.

(2) Six items assessing whether in the past 3 months alcohol had a harmful effect on respondents' (1) friendships/social life, (2) physical health, (3) outlook on life (happiness), (4) home life or marriage, (5) work, studies, or employment opportunities, or (6) financial position [68].

(3) Mediator variables, perceived drinking norms and perceived risk will be measured using the same items as on the baseline survey.

(4) Formal addictions treatment utilization will be measured using the same single item as the baseline survey, modified to ask about the last three months.

Six-month Follow-up Survey

(1) Using the same measures of alcohol consumption as the baseline survey, respondents' drinking over the time since the three-month follow-up will be assessed.

(2) The same six items assessing any harmful effects of alcohol in the past 3 months alcohol.

(3) Formal addictions treatment utilization will be measured using the same single item as the baseline survey, modified to ask about the last three months. In addition, respondents in the personalized feedback pamphlet condition will be asked if they called the telephone number provided on the pamphlet in order to assess whether the pamphlet stimulates additional help seeking.

(4) Knowledge of whether the household received any drinking-related materials will be assessed. Respondents will be asked if their household received either of the two pamphlets that were mailed. If they received one they will be asked which one and whether they read it. Those who received the "Evaluate Your Drinking" pamphlet and read it will be asked if the did the self-test contained in the pamphlet.

Collateral Confirmation

At the six-month follow-up respondents will be asked to provide the name of a collateral to confirm their drinking self-reports. Collaterals identified for participation will be mailed a letter explaining that they have been nominated to act as a collateral. Collaterals will then be contacted by telephone to confirm their willingness to talk about the respondent's drinking. The 10-minute telephone interview will cover the respondent's drinking in the past three months and any use of treatment services for alcohol problems during the past three months (mirror items of the respondents' six-month follow-up survey). Collaterals will be offered a $20 honorarium for completing this interview.

Data Analysis

Power Analyses

The power analysis conducted to estimate the sample size required to test the hypotheses of this study used the procedures suggested by Cohen [73] for estimating statistical significance. That is, what is the sample size required to detect an increase in the variance explained due to the inclusion of the personalized feedback pamphlet intervention into the model (Hypothesis 1)? Based on pilot study results, a 1% increase in explained variance can be expected (a small effect size of $f = 0.10$; note – corresponded to a reduction of two drinks per week in the pilot study). Following the convention that studies should be designed to have a statistical power of at least 80%, and that hypotheses be tested at the .05 level of significance, SamplePower 1.0 [74] was used to estimate the required sample size. These specifications resulted in a final sample (required after attrition) of $N = 390$ in each condition ($N = 1170$ total). Given that the inclusion of perceived risk as a moderator resulted in an increase in explained variance of 9% (also in the pilot test), this same sample size would result in a power of better than 99% to test for the mediator and moderator hypotheses (assuming a similar effect size). Results presented by Neighbors and colleagues [44] found similar effect sizes for the mediator hypothesis regarding perceived drinking norms and the moderator hypothesis regarding drinking for social reasons, indicating that the proposed sample size will be adequate to test these hypotheses as well.

Although Structural Equation Modeling [75], the analysis method to be used, allows for the sophisticated treatment of missing data, it is still important to assure an adequate number of respondents are followed-up. Based on previous experience [76], it is estimated that 80% of respondents will be followed-up on the six-month follow-up. This means that about 1830 respondents who agree to the follow-up will need to be recruited on the baseline survey in order to account for respondent attrition. We are assuming a worst-case scenario of an 80% follow-up rate on each of the three-month and six-month follow-up interviews but that the 20% lost on each follow-up survey will be different respondents. Thus, to obtain 1170 respondents with complete follow-up data, a follow-up rate of 80% × 80% = 64% is assumed. Given that previous research [77] has indicated that 75% of problem drinkers will agree to be followed-up, the baseline survey will need to include 2440 respondents who are problem drinkers (AUDIT score of 8 or more). As 20% of the baseline sample will be problem drinkers, the full baseline survey will need to screen 12200 respondents who consume alcohol at least once per month (based on previous experience, 20% of monthly alcohol drinkers have an AUDIT score of 8 or more). This will allow a final sample size of 1170 problem drinkers participating in the six-month follow-up. In summary, from 12,200 respondents interviewed at baseline 20% (n = 2440) will be problem drinkers, 75% of whom (n = 1830) will agree to participate in the follow-up interviews, and an 80% follow-up rate for each follow-up survey will give total data for 1170 respondents.

Analysis Plan

The analysis plan will follow the description of the use of Structural Equation Modeling (SEM) [75] for the analyses of experimental studies provided by Neighbors et al. [44] and by Russell et al. [78]. SEM with full information maximum likelihood will be used. Effect sizes will be reported [79,80].

Discussion

The primary goal of this project is to evaluate the efficacy of an ultra-brief self-help intervention for non-treatment seeking problem drinkers in the general population. One strength of the proposed study is that it merges population-based methods with a randomized controlled trial.

Thus, a general population survey will be employed using a random digit dialing method in order to recruit a good cross-section of problem drinkers from the Toronto population. Respondents will be randomly assigned to condition, allowing for causal inference about any differences observed.

The information from the proposed trial may help illuminate effective ways of promoting change among problem drinkers who do not seek formal treatment. If the project finds support for the efficacy of a pamphlet-based feedback intervention, it would provide justification to substantially increase the accessibility of this self-help method for problem drinking. This would be accomplished by sending the pamphlet directly to the public in bulk mailing, to health care settings (treatment centers, hospitals, doctors' offices), and to other social services such as unemployment agencies and welfare departments. As problem drinking is common in all these settings, a research evaluated ultra-brief intervention made freely available to all those in need would help broaden the base of treatment for alcohol problems.

Abbreviations

AUDIT: Alcohol Use Disorders Identification Test; CAMH: Centre for Addiction and Mental Health; SEM: Structural Equation Modeling.

Competing interests

The authors declare that they have no competing interests.

Authors' contributions

All authors have made an intellectual contribution to this research protocol. JAC is the Principal Investigator of the project and wrote-up the protocol. All authors have contributed to the drafting process, and all authors have read and approved the final manuscript.

Additional material

Additional file 1

An electronic copy of the intervention pamphlet, Evaluate Your Drinking, to be used in the research trial.
Click here for file
[http://www.biomedcentral.com/content/supplementary/1471-2458-8-298-S1.pdf]

Additional file 2

An electronic copy of the control condition pamphlet, Do you know .... Alcohol, to be used in the research trial.
Click here for file
[http://www.biomedcentral.com/content/supplementary/1471-2458-8-298-S2.pdf]

Acknowledgements

This study is funded by the National Institute on Alcohol Abuse and Alcoholism (NIAAA). Grant #R01 AA015680-01A2.

References

1. Institute of Medicine: *Broadening the base of treatment for alcohol problems* Washington, DC: National Academy Press; 1990.
2. Cahalan D: **Studying drinking problems rather than alcoholism.** In *Recent developments in alcoholism Volume 5*. Edited by: Galanter M. New York: Plenum; 1987:363-372.
3. Room R, Bondy S, Ferris J: **The risk of harm to oneself from drinking, Canada 1989.** *Addiction* 1995, **90:**499-513.
4. Roizen R, Cahalan D, Shanks P: **"Spontaneous remission" among untreated problem drinkers.** In *Longitudinal research on drug use: Empirical findings and methodological issues* Edited by: Kandel DB. Washington, DC: Hemisphere; 1978:197-221.
5. Sobell LC, Sobell MB, Toneatto T: **Recovery from alcohol problems without treatment.** In *Self-control and the addictive behaviours* Edited by: Heather N, Miller WR, Greeley J. New York, NY: Maxwell MacMillan; 1992:198-242.
6. Hasin DS: **Treatment/self-help for alcohol-related problems: Relationship to social pressure and alcohol dependence.** *J Stud Alcohol* 1994, **55(6):**660-666.
7. Burton TL, Williamson DL: **Harmful effects of drinking and the use and perceived effectiveness of treatment.** *J Stud Alcohol* 1995, **56:**611-615.
8. Cunningham JA, Breslin FC: **Only one in three people with alcohol abuse or dependence ever seek treatment.** *Addict Behav* 2004, **29(1):**221-223.
9. Dawson DA, Grant BF, Stinson FS, Chou PS, Huang B, Ruan WJ: **Recovery from DSM-IV alcohol dependence: United States, 2001–2002.** *Addiction* 2005, **100(3):**281-292.
10. Cunningham JA, Sobell LC, Sobell MB, Agrawal S, Toneatto T: **Barriers to treatment: Why alcohol and drug abusers delay or never seek treatment.** *Addict Behav* 1993, **18:**347-353.
11. Roizen R: *Barriers to alcoholism treatment* Berkeley, CA: Alcohol Research Group; 1977.
12. Tuchfeld BS: *Changes in patterns of alcohol use without the aid of formal treatment: An exploratory study of former problem drinkers* Research Triangle Park, NC: Research Triangle Institute; 1976.
13. Grant BF: **Barriers to alcoholism treatment: Reasons for not seeking treatment in a general population sample.** *J Stud Alcohol* 1997, **58:**365-371.
14. Breslin FC, Sobell MB, Sobell LC, Buchan G, Cunningham JA: **Toward a stepped care approach to treating problem drinkers: The predictive utility of within treatment variables and therapist prognostic ratings.** *Addiction* 1997, **92(11):**1479-1489.
15. Sobell MB, Sobell LC: **Treatment for problem drinkers: A public health priority.** In *Addictive Behaviors Across the Life Span* Edited by: Baer JS, Marlatt GA, McMahon RJ. Newbury Park, NJ: Sage Publications; 1993:138-157.
16. Abrams DB, Orleans CT, Niaura RS, Goldstein MG, Prochaska JO, Velicer W: **Integrating individual and public health perspectives for treatment of tobacco dependence under managed health care: A combined stepped care and matching model.** *Ann Behav Med* 1996, **18:**290-304.
17. Abrams DB, Clayton R: **Transdisciplinary research to improve brief interventions for addictive behaviors.** In *Adolescents, alcohol, and substance abuse* Edited by: Monti PM, Colby TA, O'Leary TA. New York: Guilford; 2001:321-341.
18. World Health Organization: **World Health Report 2002: Reducing risks, promoting health life.** Geneva: World Health Organization; 2002.
19. Babor TF, Caetano R, Casswell S, Edwards G, Giesbrecht N, Graham K, Grube JW, Gruenwald P, Hill P, Holder H, *et al.*: *Alcohol: No Ordinary Commodity – Research and Public Policy* Oxford and London: Oxford University Press; 2003.
20. Stockwell T, Gruenwald P, Toumbourou JW, Loxley W, (Eds): *Preventing harmful substance use: The evidence base for policy and practise* Toronto: John Wiley and Sons; 2005.
21. Bien TH, Miller WR, Tonigan JS: **Brief interventions for alcohol problems: A review.** *Addiction* 1993, **88:**315-336.
22. Miller WR: **Rediscovering fire: Small interventions, large effects.** *Psychol Addict Behav* 2000, **14:**6-18.

23. Walters ST, Neighbors C: **Feedback interventions for college alcohol misuse: what, why and for whom?** *Addict Behav* 2005, **30(6):**1168-1182.
24. Room R, Babor T, Rehm J: **Alcohol and public health.** *Lancet* 2005, **365(9458):**519-530.
25. National Institute on Alcohol Abuse and Alcoholism: *Helping patients who drink too much: A clinician's guide* Rockville, MD: National Institute on Alcohol Abuse and Alcoholism; 2005.
26. Denny CH, Serdula MK, Holtzman D, Nelson DE: **Physician advice about smoking and drinking: are U.S. adults being informed?** *Am J Prev Med* 2003, **24(1):**71-74.
27. Friedmann PD, McCullough D, Chin MH, Saitz R: **Screening and intervention for alcohol problems. A national survey of primary care physicians and psychiatrists.** *J Gen Intern Med* 2000, **15(2):**84-91.
28. Cunningham JA, Humpheys K, Kypri K, van Mierlo T: **Formative evaluation and three-month follow-up of an online personalized assessment feedback intervention for problem drinkers.** *J Med Internet Res* 2006, **8(2):**e5.
29. Cunningham JA, Selby PL, Kypri K, Humphreys KN: **Access to the Internet among drinkers, smokers and illicit drug users: Is it a barrier to the provision of interventions on the World Wide Web?** *Med Inform Internet Med* 2006, **31(1):**53-58.
30. Horrigan J, Raine L: **When facing a tough decision, 60 million Americans now seek the Internet's help – the Internet's growing role in life's major moments.** *Pew Internet & American Life Project* 2006 [http://pewresearch.org/obdeck/?ObDeckID=19].
31. Fox S: **Health Information Online: Eight in ten Internet users have looked for health information online.** *Pew Internet and American Life Project* 2005 [http://www.pewinternet.org/pdfs/PIP_Healthtopics_May05.pdf].
32. Agostinelli G, Brown JM, Miller WR: **Effects of normative feedback on consumption among heavy drinking college students.** *J Drug Educ* 1995, **25(1):**31-41.
33. Sobell LC, Cunningham JA, Sobell MB, Agrawal S, Gavin DR, Leo GI, Singh KN: **Fostering self-change among problem drinkers: A proactive community intervention.** *Addict Behav* 1996, **21(6):**817-833.
34. Agostinelli G, Miller WR: **Drinking and thinking: How does personal drinking affect judgments of prevalence and risk.** *J Stud Alcohol* 1994, **55:**327-337.
35. Neighbors C, Lewis MA, Bergstrom RL, Larimer ME: **Being controlled by normative influences: self-determination as a moderator of a normative feedback alcohol intervention.** *Health Psychol* 2006, **25(5):**571-579.
36. Lewis MA, Neighbors C: **Optimizing personalized normative feedback: the use of gender-specific referents.** *J Stud Alcohol Drugs* 2007, **68(2):**228-237.
37. Larimer ME, Lee CM, Kilmer JR, Fabiano PM, Stark CB, Geisner IM, Mallett KA, Lostutter TW, Cronce JM, Feeney M, *et al.*: **Personalized mailed feedback for college drinking prevention: a randomized clinical trial.** *J Consult Clin Psychol* 2007, **75(2):**285-293.
38. Wild TC, Cunningham JA, Roberts AB: **Controlled study of brief personalized assessment-feedback for drinkers interested in self-help.** *Addiction* 2007, **102(2):**241-250.
39. Cunningham JA, Wild TC, Bondy SJ, Lin E: **Impact of normative feedback on problem drinkers: A small-area population study.** *J Stud Alcohol* 2001, **62:**228-233.
40. Miller WR, Sovereign RG, Krege B: **Motivational interviewing with problem drinkers: II. The Drinker's Check-up as a preventive intervention.** *Behavioural Psychotherapy* 1988, **16:**251-268.
41. Borsari B, Carey KB: **Effects of a brief motivational intervention with college student drinkers.** *J Consult Clin Psychol* 2000, **68(4):**728-733.
42. Murphy JG, Duchnick JJ, Vuchinich RE, Davison JW, Karg RS, Olson AM, Smith AF, Coffey TT: **Relative efficacy of a brief motivational intervention for college student drinkers.** *Psychol Addict Behav* 2001, **15:**373-379.
43. Baer JS, Kivlahan DR, Blume AW, McKnight P, Marlatt GA: **Brief intervention for heavy-drinking college students: 4-year follow-up and natural history.** *Am J Public Health* 2001, **91:**1310-1316.
44. Neighbors C, Larimer ME, Lewis MA: **Targeting misperceptions of descriptive drinking norms: efficacy of a computer-delivered personalized normative feedback intervention.** *J Consult Clin Psychol* 2004, **72(3):**434-447.
45. Collins SE, Carey KB, Sliwinski MJ: **Mailed personalized normative feedback as a brief intervention for at-risk college drinkers.** *J Stud Alcohol* 2002, **63:**559-567.
46. Lewis MA, Neighbors C: **Social norms approaches using descriptive drinking norms education: a review of the research on personalized normative feedback.** *J Am Coll Health* 2006, **54(4):**213-218.
47. Walters ST, Bennet ME, Miller JH: **Reducing alcohol use in college students: A controlled trial of two brief interventions.** *J Drug Educ* 2000, **30:**361-372.
48. Geisner IM, Neighbors C, Larimer ME: **A randomized clinical trial of a brief, mailed intervention for symptoms of depression.** *J Consult Clin Psychol* 2006, **74(2):**393-399.
49. Carey KB, Scott-Sheldon LA, Carey MP, DeMartini KS: **Individual-level interventions to reduce college student drinking: a meta-analytic review.** *Addict Behav* 2007, **32(11):**2469-2494.
50. Kanfer FH: **Implications of a self-regulation model of therapy for treatment of addictive behaviors.** In *Treating addictive behaviors: Processes of change* Edited by: Miller WR, Heather N. New York: Plenum; 1986.
51. Agostinelli G, Floyd TE, Grube JW, Woodall G, Miller JH: **Alcohol problem recognition as a function of own and others' perceived drinking.** *Addict Behav* 2004, **29(1):**143-157.
52. Neal DJ, Carey KB: **Developing discrepancy within self-regulation theory: use of personalized normative feedback and personal strivings with heavy-drinking college students.** *Addict Behav* 2004, **29(2):**281-297.
53. Borsari B, Carey KB: **Peer influences on college drinking: a review of the research.** *J Subst Abuse* 2001, **13(4):**391-424.
54. Neighbors C, Dillard AJ, Lewis MA, Bergstrom RL, Neil TA: **Normative misperceptions and temporal precedence of perceived norms and drinking.** *J Stud Alcohol* 2006, **67(2):**290-299.
55. Becker MH, Rosenstock IM: **Comparing social learning theory and the health belief model.** In *Advances in health education and promotion Volume 2*. Edited by: Ward WB. Greenwich, CT: JAI Press; 1987:245-249.
56. Rogers RW: **Cognitive and physiological processes in fear appeals and attitude change: A revised theory of protection motivation.** In *Social Psychophysiology* Edited by: Cacioppo J, Petty R. New York: Guilford; 1983:153-176.
57. Ajzen I, Fishbein M: *Understanding attitudes and predicting behavior* Englewood Cliffs, NJ: Prentice-Hall; 1980.
58. Ronis DL: **Conditional health threats: Health beliefs, decisions, and behaviors among adults.** *Health Psychol* 1992, **11:**127-134.
59. Weinstein ND: **Testing four competing hypotheses of health-protective behavior.** *Health Psychol* 1993, **12:**235-245.
60. Wild TC, Cunningham JA: **Psychosocial determinants of perceived vulnerability to harm among adult drinkers.** *J Stud Alcohol* 2001, **62:**105-113.
61. Leigh BC: **In search of the seven dwarves: Issues of measurement and meaning in alcohol expectancy research.** *Psychol Bull* 1989, **105:**361-373.
62. Stacy AW, Widaman KF, Marlatt GA: **Expectancy models of alcohol use.** *J Pers Soc Psychol* 1990, **58(5):**918-928.
63. Cooper ML: **Motivations for alcohol use among adolescents: Development and validation of a four-factor model.** *Psychol Assess* 1994, **6:**117-128.
64. Babor TF, De La Fuente MF, Saunders JB, Grant M: *AUDIT – The alcohol use disorders identification test: Guidelines for use in primary health care* Geneva, Switzerland: World Health Organization; 1989.
65. Saunders JB, Conigrave KM: **Early identification of alcohol problems.** *Cmaj* 1990, **143:**1060-1069.
66. Brown JM, Miller WR: **Impact of motivational interviewing on participation and outcome in residential alcoholism treatment.** *Psychol Addict Behav* 1993, **7(4):**211-218.
67. Adlaf EM, Ialomiteanu A, Paglia A: **CAMH Monitor 1999: Technical Guide.** Toronto: Centre for Addiction and Mental Health; 1999.
68. Statistics Canada: *Microdata User's Guide: Canada's Alcohol and Other Drugs Survey* Ottawa, Canada: Statistics Canada, Special Surveys Division; 1994.
69. Baer JS, Stacy A, Larimer M: **Biases in the perception of drinking norms among college students.** *J Stud Alcohol* 1991, **52(6):**580-586.

70. Wild TC, Cunningham JA: **Alcohol-related risk perceptions: A multimethod study.** *Kettil Bruun Society for Social and Epidemiological Research on Alcohol: June, 2008; Victoria, Canada* 2008.
71. Fromme K, Stroot E, Kaplan D: **Comprehensive effects of alcohol: Development and psychometric assessment of a new expectancy questionnaire.** *Psychol Assess* 1993, **5:**19-26.
72. Grant BF, Peterson A, Dawson DS, Chou SP: *Source and accuracy statement for the National Longitudinal Alcohol Epidemiologic Survey* Rockville, MD: National Institute on Alcohol Abuse and Alcoholism; 1994.
73. Cohen J, Cohen P: *Applied multiple regression/correlation analysis for the behavioral sciences* 2nd edition. Hillsdale, NJ: Erlbaum; 1983.
74. Borenstein M, Rothstein H, Cohen J: **SamplePower.** Chicago: SPSS Inc; 1997.
75. Kline RB: *Principles and Practice of Structural Equation Modeling* New York: The Guilford Press; 1998.
76. Cunningham JA, Koski-Jännes A, Wild TC, Cordingley J: **Treating alcohol problems with self-help materials: A population study.** *J Stud Alcohol* 2002, **63:**649-654.
77. Wild TC, Cunningham JA, Adlaf E: **Nonresponse in a follow-up to a representative telephone survey of adult drinkers.** *J Stud Alcohol* 2001, **62:**257-261.
78. Russell DW, Kahn JH, Spoth R, Altmaier EM: **Analyzing data from experimental studies: A latent variable structural equation modeling approach.** *J Couns Psychol* 1998, **45:**18-29.
79. Rosenthal R, Rosnow RL: *Essentials of behavioral research: Methods and data analysis* 2nd edition. New York: McGraw Hill; 1991.
80. Cohen J: **A power primer.** 1992, **112:**155-159.

Pre-publication history

The pre-publication history for this paper can be accessed here:

http://www.biomedcentral.com/1471-2458/8/298/prepub

Publish with **BioMed Central** and every scientist can read your work free of charge

"BioMed Central will be the most significant development for disseminating the results of biomedical research in our lifetime."

Sir Paul Nurse, Cancer Research UK

Your research papers will be:

- available free of charge to the entire biomedical community
- peer reviewed and published immediately upon acceptance
- cited in PubMed and archived on PubMed Central
- yours — you keep the copyright

Submit your manuscript here:
http://www.biomedcentral.com/info/publishing_adv.asp

BioMedcentral
