# Supplementary figures and images for: Ultra-Brief Intervention for Problem Drinkers: Results from a Randomized Controlled Trial
Source: PLoS One. 2012 Oct 24;7(10):e48003. doi: 10.1371/journal.pone.0048003 (PMC3480504; doi:10.1371/journal.pone.0048003)

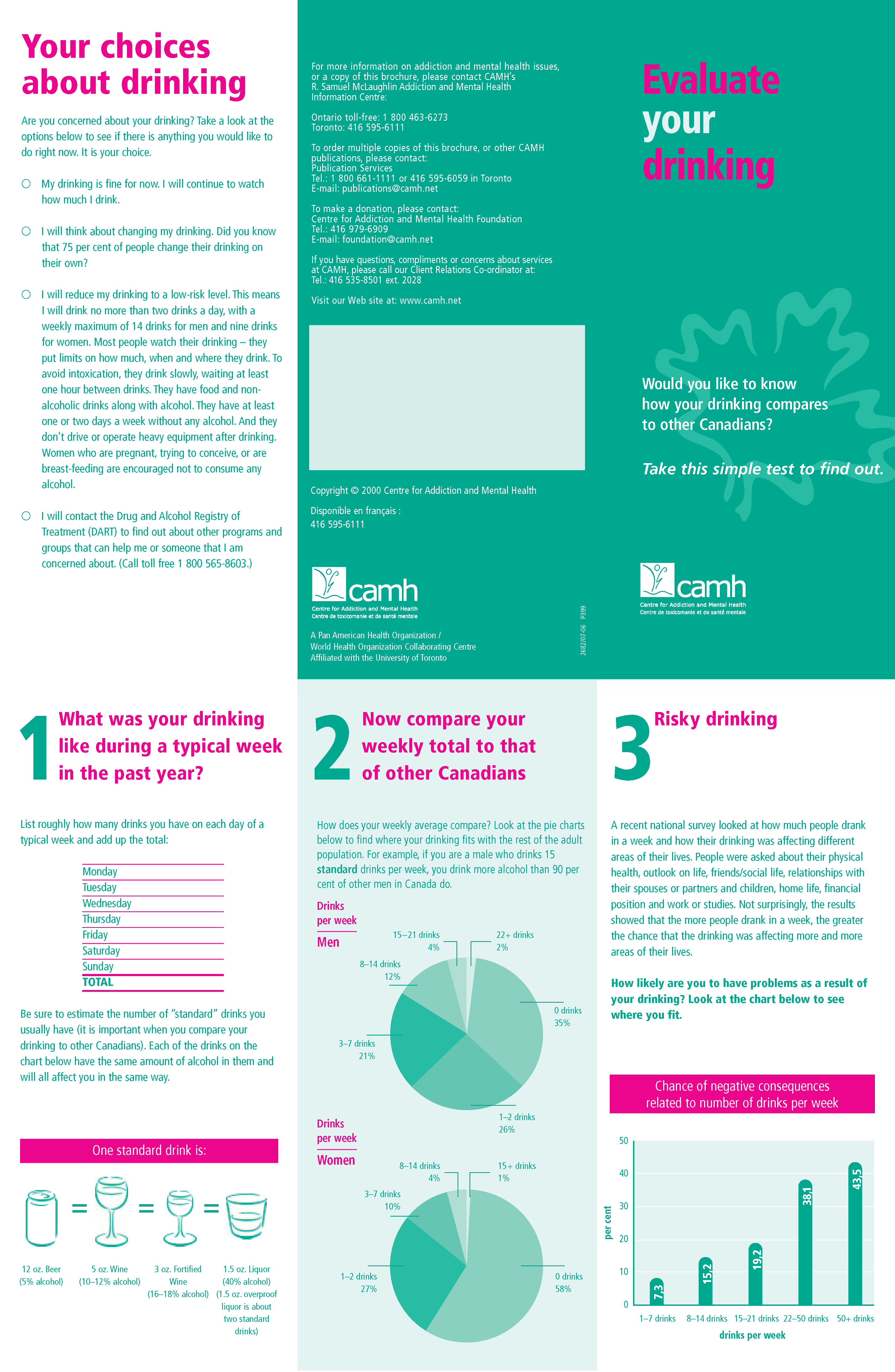

Supplement: Appendix S1 — Copy of Evaluate Your Drinking Pamphlet. (TIF) [file pone.0048003.s003.tif]

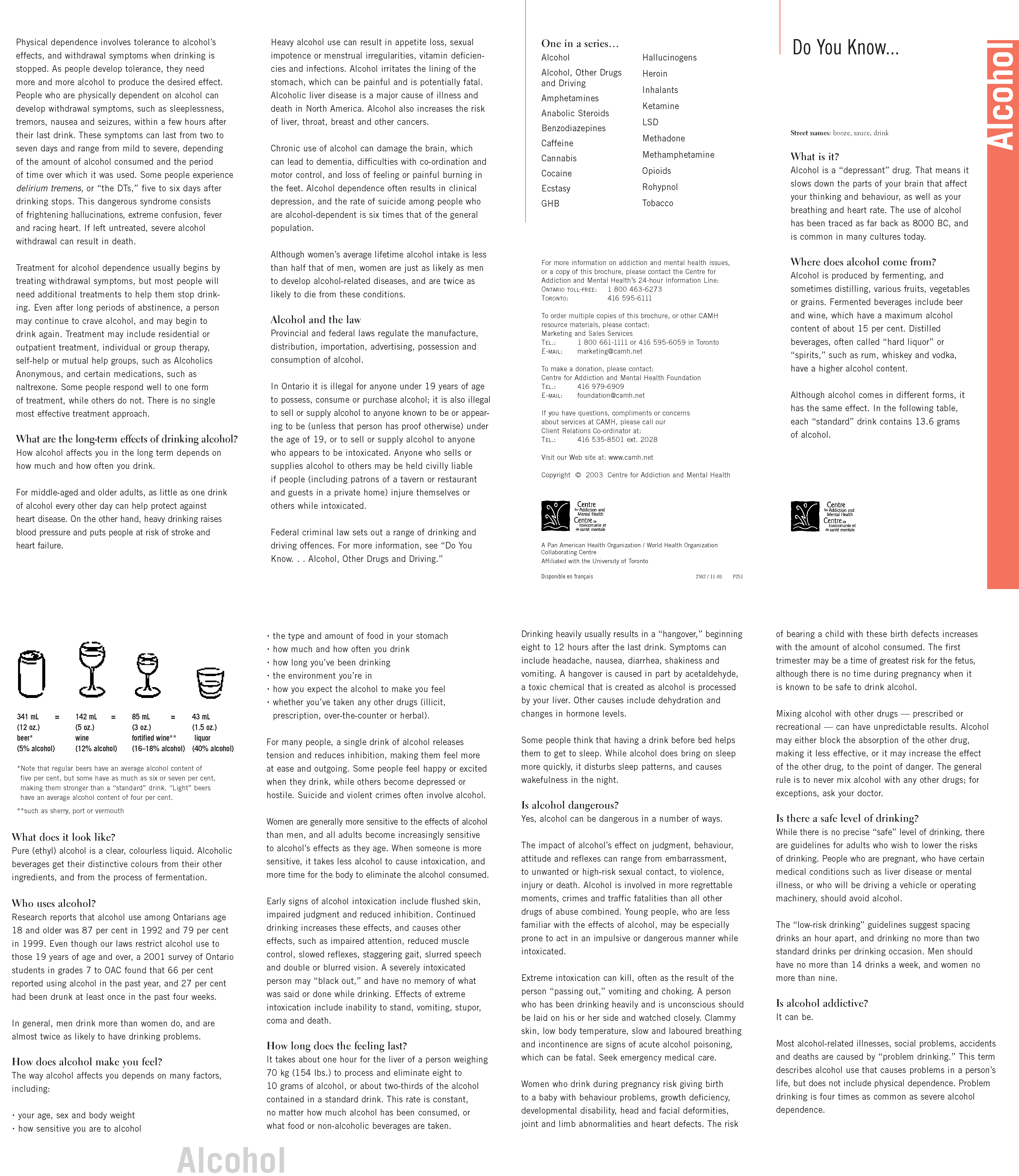

Supplement: Appendix S2 — Copy of Control Pamphlet. (TIF) [file pone.0048003.s004.tif]
